# Acute hepatitis B virus infection despite vaccination in a patient treated by infliximab: a case report

**DOI:** 10.1186/s12876-022-02397-5

**Published:** 2022-06-29

**Authors:** Juliette Besombes, Faouzi Souala, Guillaume Bouguen, Dominique Guyader, Claire Grolhier, Vincent Thibault, Charlotte Pronier

**Affiliations:** 1 Department of Virology, INSERM, EHESP, IRSET - UMR_S 1085, Pontchaillou University Hospital, Univ Rennes, Rennes, France; 2 Infectious Diseases and Intensive Care Unit, Pontchaillou University Hospital, Rennes, France; 3 Department of Hepato-Gastroenterology, UMR 991, Pontchaillou University Hospital, Univ Rennes, Rennes, France; 4 Department of Liver Diseases (SMF), UMR 1241, Pontchaillou University Hospital, Univ Rennes, Rennes, France

**Keywords:** Hepatitis B, Immunocompromised, Vaccination, Immune escape, Infliximab, Acute hepatitis

## Abstract

**Background:**

Despite an effective vaccine, hepatitis B remains a major global health problem due to its significant morbidity and mortality. Vaccination in immunosuppressed patients such as those treated for an inflammatory bowel disease (IBD) can be less effective. This case describes an uncommon original diagnosis of an acute hepatitis B infection occurring in a vaccinated but immunocompromised IBD patient under long-term infliximab treatment. A low anti-HBs titer and the presence of HBsAg escape mutations are possible hypotheses to explain this unexpected infection.

**Case presentation:**

A 28-year-old Caucasian male, regularly followed-up for a Crohn’s disease treated by infliximab, was regularly screened for sexually transmissible infections because of at-risk behaviors. Despite a correct immunization scheme against hepatitis B virus (HBV), an active HBV infection was diagnosed during one of those screenings. Retrospective testing of a sample collected 6 months earlier was in favor of an evolution from an acute hepatitis B toward a chronic hepatitis B. The patient has always had a low anti-HBs antibody levels (near the threshold of 10 IU/L) possibly explaining his infection. In addition, HBV sequencing revealed a genotype A2 HBV strain, carrying the sD144A substitution on the S protein, known as a potential immune escape variant. Dual therapy combining tenofovir disoproxil fumarate and emtricitabine, active against HBV but also efficient as an HIV pre-exposure prophylaxis, was initiated. Ten months after treatment initiation, all surrogate biochemical and virological endpoints for HBV functional cure were achieved. Treatment and periodical monitoring are being maintained.

**Conclusion:**

Emphasis should be placed on HBV screening, vaccination and regular monitoring of patients under long-term immunosuppressive therapy, particularly those with at-risk behaviors.

## Background

Hepatitis B is a viral infection efficiently prevented by a safe and effective vaccine. Most of healthy adults with acute hepatitis B spontaneously resolve the infection as a result of an efficient and timely innate and adaptive immune responses [1]. Yet, any immunosuppressive condition may jeopardize such spontaneous recovery. In patients undergoing immunosuppressive therapy, the risks of infection or reactivation largely depend on the type of administered drugs [2]. Thus, for non-immunized patients, vaccination against hepatitis B virus (HBV) is recommended before introduction of any immunosuppressive therapy [2, 3]. Here, we describe a case of a likely acute HBV infection occurring in a patient with an inflammatory bowel disease (IBD) treated by infliximab and vaccinated in childhood, years prior to his immunosuppressive treatment initiation.

## Case presentation

A 28-year-old Caucasian male was regularly followed-up for a Crohn’s disease (CD) treated by infliximab, an anti-tumor necrosis factor (TNF) therapy. Infliximab treatment had been initiated in August 2011 and maintained up to now. In 1997, he received a complete vaccination scheme of 3 doses of Engerix-B10 (0–1–6 months). In September 2010, a CD was diagnosed. In June 2011, serological HBV screening performed before infliximab initiation revealed the presence of a low anti-HBs titer of 15.4 IU/L without HBs antigen (HBsAg) (Table 1). At that time, the anti-HBc status was not determined. In April 2019, the HBV serology confirmed a protective anti-HBs antibodies (Ab) titer at 15 IU/L and the absence of anti-HBc Ab; a serological profile indicative of an efficient vaccine induced immunity. In parallel of his CD management, this patient, who had a high-risk of acquiring sexually transmissible infections (STI), has been receiving regular STI screenings at a free sexual health center. In June 2020, HCV and HIV serologies were negative while the syphilis serology showed an inconclusive profile (Table 1). The HBV serological markers revealed an ongoing hepatitis B infection as documented by positive HBsAg and anti-HBc Ab but an absence of anti-HBs Ab. Liver function tests indicated a moderate hepatic cytolysis, with ALT around 4 times the upper limit of normal values. Additional hepatitis B markers were reactive for anti-HBc IgM, HBeAg and viral replication reaching 8 log IU/mL. Hepatitis Delta testing was negative. Interestingly, through retrospective testing of a sample collected 6 months earlier, the serological profile was already in agreement with an active HBV infection. HBV sequencing identified a genotype A2 strain carrying the sD144A substitution on the S protein. In July 2020, 1 month after HBV diagnosis and 7 months after the first evidence of a positive HBsAg, persistence of HBsAg and viral replication were in favor of a transition toward a chronic HBV infection. The FibroTest® result (F0–F1) did not support a significant fibrosis. To prevent the risk of HBV transmission and the development of liver lesions, a treatment by tenofovir disoproxil fumarate (TDF) and emtricitabine, active against HBV and also efficient as an HIV pre-exposure prophylaxis (PrEP), was initiated in September 2020. Five months after treatment initiation, HBeAg remained positive but a decline in HBV-DNA and HBsAg levels was recorded. Twelve months after treatment initiation, all surrogate virological endpoints for HBV functional cure were achieved. ALT levels remained slightly elevated while HBe seroconversion was observed. HBsAg became undetectable by both current and ultrasensitive HBsAg assays without anti-HBs seroconversion. HBV-DNA remained slightly detectable but below the limit of quantification of 10 IU/ml (Table 1). On the last control, 18 months after treatment initiation, HBV-DNA was undetectable in the blood and anti-HBs Ab appeared. However, ALT levels had increased again (1.8 ULN). Antiviral treatment and periodical STI monitoring are being maintained, in front of high-risk behaviors and to ascertain durable HBsAg loss.

**Table 1:** Outcome of patient’s biological analyses over several follow-ups

	June 2011 Pre-infliximab evaluation	April 2019	January 2020 M-6	June 2020 HBV infection Diagnosis D0	July 2020 M + 1	February 2021	September 2021	March 2022
						Under HBV treatment (Since 09/2020)		
						M + 8	M + 15	M + 21
Place of consultation	Gastroenterology	General practitioner	CeGIDD	CeGIDD	Infectious disease	Infectious disease	Infectious disease	Infectious disease
Infliximab treatment	−	+	+	+	+	+	+	+
Serology and HBV markers								
HBV								
HBsAg	Negative	Negative	Positive*	Positive	Positive	ND	ND	ND
HBsAg (IU/mL)	ND	ND	Positive (3.3)*	Positive (18,000)*	Positive (18,000)	Positive (5,100)	Negative (< 0.03)	Negative (< 0.03)
Anti-HBs Ab (IU/L)	Positive (15.4)	Positive (15)	Negative (5.3)	Negative (< 2)	Negative (< 2)	Negative (< 2)	Negative (8.3)	Positive (17.4)
Anti-HBc Ab	ND	Negative	Negative*	Positive	Positive	ND	ND	ND
Anti-HBc IgM Ab	ND	ND	Negative*	Positive*	Positive	ND	ND	ND
HBeAg	ND	ND	Positive*	Positive*	Positive	Positive	Negative	Negative
Anti-HBe Ab	ND	ND	Negative*	Negative*	Negative	Negative	Positive	Positive
HBV-DNA VL (log_10_ IU/mL)	ND	ND	5.0*	8.0*	8.2	3.84	< 1 Detected HBV-DNA	< 1 Not detected HBV-DNA
Anti-HDV Ab	ND	ND	ND	Negative*	Negative	Negative	ND	ND
Anti-HCV Ab	Negative	ND	Negative	Negative	Negative	Negative	ND	Negative
Anti-HAV	ND	ND	ND	ND	Negative	Positive	ND	ND
Anti-HIV	ND	ND	Negative	Negative	Negative	Negative	Negative	Negative
Syphilis	ND	ND	ND	Prior syphilis treated or untreated, early syphilis or false positive		Negative	Negative	Negative
Liver function tests								
ALT (ULN)	ND	ND	ND	4.2	3.8	3.4	1.1	1.8
AST (ULN)	ND	ND	ND	2.8	2	1.8	1	1.3

Qualitative HBs Antigen (Roche Cobas® Elecsys HBsAg II), Quantitative HBs Antigen (DiaSorin LIAISON® XL Murex HBsAg Quant), anti-HBs Antibodies (Roche Cobas® Elecsys Anti-HBs II), anti-HBc antibodies (Roche Cobas® Elecsys Anti-HBc II), anti-HBc antibodies IgM (Roche Cobas® Elecsys Anti-HBc IgM and DiaSorin LIAISON® XLHBc IgM), HBe Antigen and Antibodies (Roche Cobas® Elecsys HBeAg and anti-HBe). HBV viral load (HBV-VL) was determined by PCR (Xpert HBV viral load; Cepheid). Anti-HCV, HAV and HIV antibodies were detected using Elecsys anti-HCV II, anti-HAV II and HIV combi PT assays respectively (Roche Cobas®). Anti-HDV and syphilis antibodies were detected using Murex anti-HDV and Treponema screen assays respectively (DiaSorin LIAISON® XL)

*ULN* times over the upper limit of normal range. CeGIDD: free center for information, screening and diagnosis of HIV and STI; *ND* not determined

*Analyses added retrospectively after the HBV diagnosis in June 2020

## Discussion and conclusion

This original case reports an acute HBV infection, with a putative escape variant, despite a full HBV vaccination, in an immunocompromised patient treated with infliximab [4]. Different practitioners both for his digestive disease and for his at-risk sexual behavior regularly followed the patient. His acute HBV infection was diagnosed in favor of regular STI screening visits. The retrospective analysis of a sample collected 6 months earlier indicated the evolution of this acute hepatitis B toward a chronic hepatitis B as documented by the persistence over 6 months of the HBsAg. Any immunosuppressive condition, either acquired or treatment-induced, is associated with a risk of HBV chronicity or reactivation. This is particularly observed during inhibition of TNF-α-mediated biological effects [1, 5]. The mechanism is not fully explained but TNF-α and related cytokines are involved both in the regulation of the adaptive immune system responsible for HBV immune surveillance and the control of covalently closed circular DNA transcriptional activity [6]. While cases of HBV reactivation under infliximab therapy for chronic inflammatory diseases have already been described, our clinical case indicates that these treatments may also be associated with an impaired vaccine efficacy and the risk of an evolution toward chronicity [7, 8]. The weakened immune system may also explain the delayed appearance of anti-HBc IgM that were negative on the first HBsAg positive serum. However, the lack of anti-HBc IgM can also be observed at the very early stage of acute HBV infection, at the very onset of liver cytolysis.

Screening for hepatitis B before starting an immunosuppressive therapy is widely recommended to prevent any risk of viral reactivation in infected patients (HBsAg positive) or those with past-infection serological imprint (*i.e.* anti-HBc positive) [3, 6]. In anti-HBc positive patients, antiviral prophylaxis can be proposed particularly for treatments at high risk of reactivation [3, 9]. Non-immunized patients should be proposed vaccination at the time of IBD diagnosis and before initiating immunomodulatory treatment [2, 10]. Yet, this patient benefited from a correct full vaccine scheme a few years earlier as reported in his medical file. In addition, stable anti-HBs titers were documented from 2011 to 2019. As only HBsAg and anti-HBs Ab had been tested before the initiation of infliximab treatment in our patient, it was not possible to formally conclude on a past infection or a vaccine serological status. Indeed, the first available anti-HBc testing was performed in 2019, when the patient was already receiving infliximab. The possibility that the immunosuppressive treatment had decreased anti-HBc to undetectable level cannot be formally ruled out [11]. Yet, anti-HBs had remained at similar levels as those detected earlier and the patient had no history of clinically significant conditions related to his immunosuppression. The need to propose a reinforced vaccine boost strategy in such patient should probably be discussed especially when no proof of a high level of immunity following vaccination (anti-HBs > 100 IU/L) exists. Early vaccination, before immunosuppressive treatment initiation is preferable as the HBV vaccine response rate in patients using immunomodulators is dramatically decreased, in particular in infliximab-users, even with high dose vaccine [12, 13]. An anti-HBs titer above 10 IU/L such as recommended in healthy individuals may not be sufficient for IBD patient; the definition of a minimal threshold in such situation would be useful [10]. Thus, efforts should be made to vaccinate IBD patients against HBV or to give a booster dose to maintain high anti-HBs Ab levels before immune suppressive therapy. Under such treatments, periodical monitoring of anti-HBs titers, to ascertain efficacy, should also be promoted especially in patients at high risk of acquiring hepatitis B. Interestingly, sequencing of the HBV strain revealed a known HBsAg escape variant (sD144A), that may also have contributed to a reduced vaccine induced Ab neutralization potency [4]. This substitution, along with others located within the ‘a’ determinant (residues 124–147), are known as vaccine escape substitutions that may also jeopardize HBsAg detection by assays [14]. Fortunately, these substitutions do not impair efficacy of current nucleoside/nucleotide analogues used for HBV treatment [15]. In front of a high viral load in an immunocompromised patient, the clinical staff decided to initiate a treatment by a TDF-emtricitabine (Truvada®) association, that also comply with an HIV PrEP strategy considering the patient’s at-risk behavior [3]. One year after treatment initiation, the patient achieved HBeAg and HBsAg losses, considered for this latter as the hallmark of functional cure. Considering the undetectable HBV viral load and the functional cure serological profile, persisting subnormal ALT levels were attributed to a possible infliximab side effect.

In conclusion, this case highlights some difficulties encountered in patients under long-term immunosuppressive therapy, particularly those with at-risk behaviors. This case conveys several messages:

- A full screening based on detection of the three HBV markers (HBsAg, anti-HBs Ab, anti-HBc Ab) shoud be performed before starting an immunosuppressive treatment. Anti-HBc Ab are essential to establish a history of previous HBV infection linked to a risk of reactivation;
- Vaccination should be promoted and vaccine efficacy controlled to consider a potential booster dose in patients with anti-HBs titer less than 100 IU/L before starting an immunosuppressive therapy [9];
- Patients who are at-risk of infection need a recurrent follow-up in order to prevent and to early diagnose any infection. Special attention should be paid to patients wandering between different practitioners and those who are particularly vulnerable.

## Data Availability

The datasets used and/or analysed during the current study are available from the corresponding author on reasonable request.

## Abbreviations

Ab: Antibody
Ag: Antigen
ALT: Alanine aminotransferase
CD: Crohn’s disease
HBV: Hepatitis B virus
HIV: Human immunodeficiency virus
IBD: Inflammatory bowel disease
IU: International unit
PrEp: Pre-exposure prophylaxis
STI: Sexually transmissible infections
TDF: Tenofovir disoproxil fumarate
TNF: Tumor necrosis factor
